# Applications of Single-Cell RNA Sequencing in Cardiovascular Research

**DOI:** 10.3389/fcell.2021.810232

**Published:** 2022-01-31

**Authors:** Yu Fan, Han Zhou, Xuexue Liu, Jingyan Li, Ke Xu, Xiaodong Fu, Lei Ye, Guang Li

**Affiliations:** ^1^ Key Laboratory of Medical Electrophysiology, Ministry of Education and Medical Electrophysiological Key Laboratory of Sichuan Province, Collaborative Innovation Center for Prevention of Cardiovascular Diseases, Institute of Cardiovascular Research, Southwest Medical University, Luzhou, China; ^2^ Department of Obstetrics, Sichuan Clinical Research Center for Birth Defects, The Affiliated Hospital of Southwest Medical University, Luzhou, China; ^3^ National Heart Research Institute of Singapore, Singapore, Singapore

**Keywords:** single-cell RNA sequencing, cellular heterogeneity, cardiovascular system, therapeutic target, cell based therapy

## Abstract

In recent years, cardiovascular disease (CVD) continues to be the leading cause of global disease burden. Extensive efforts have been made across basic, translational, and clinical research domains to curb the CVD epidemic and improve the health of the population. The successful completion of the Human Genome Project catapulted sequencing technology into the mainstream and aroused the interests of clinicians and scientific researchers alike. Advances in single-cell RNA sequencing (scRNA-seq), which is based on the transcriptional phenotypes of individual cells, have enabled the investigation of cellular fate, heterogeneity, and cell–cell interactions, as well as cell lineage determination, at a single-cell resolution. In this review, we summarize recent findings on the embryological development of the cardiovascular system and the pathogenesis and treatment of cardiovascular disease, as revealed by scRNA-seq technology. In particular, we discuss how scRNA-seq can help identify potential targets for the treatment of cardiovascular diseases and conclude with future perspectives for scRNA-seq.

## Introduction

Cardiovascular disease (CVD), primarily ischemic heart disease and stroke, remains the leading cause of mortality and morbidity across the world (GBD 2019 Diseases and Injuries Collaborators, 2020). Recent evidence indicates that the prevalence of total CVD has continued to increase, nearly doubling from 271 million in 1990 to 523 million in 2019. Similarly, the number of CVD-related deaths has steadily increased from 12.1 million in 1990 reaching 18.6 million in 2019. The burden of CVD continues its decade-long rise in almost all countries except high-income countries. In most low- and middle-income countries, the overall burden of CVD, in terms of morbidity and mortality, is higher than that in high-income countries (Roth et al., 2020). Therefore, the promotion of cardiovascular health remains an important task for clinicians and scientific researchers.

With the completion of the Human Genome Project and the rapid development of sequencing technologies, there has been a growing interest in single-cell genomic and transcriptomic studies. Single-cell sequencing investigates biodiversity and heterogeneity at a single-cell resolution (Grün and van Oudenaarden, 2015). Since it was rated as “The Method of the Year” by Nature Methods in 2013, multiple single-cell sequencing methods and platforms have been developed for various applications. In general, single-cell genome sequencing is used to detect copy number variations and single-nucleotide variations within the genome of individual cells (Adey, 2021). Similarly, single-cell epigenome sequencing is used to detect modifications, such as methylation of DNA and histones, in the epigenome of individual cells (Prompsy et al., 2020).

In contrast, single-cell RNA sequencing (scRNA-seq) is used to detect the mRNA level of individual cells (Paik et al., 2020). In the field of modern cardiovascular research, it is a powerful tool for elucidating cellular heterogeneity, cell types, intercellular crosstalk, and trajectory analysis of cellular dynamics (Litviňuková et al., 2020; Cheng et al., 2021; Grancharova et al., 2021; Kan et al., 2021). In this review, we summarize published research using scRNA-seq on the embryological development of the cardiovascular system and pathogenesis of CVD. This is followed by a discussion on how progress in scRNA-seq technology can enable the identification of new targets for cell-based therapy. In addition, we compare the differences and similarities between scRNA-seq and bulk RNA sequencing and discuss the potential applications of scRNA-seq in cardiovascular research.

### scRNA-Seq and Cardiovascular Development

Compared to adult hearts, embryonic and neonatal cardiac tissues are easier to digest and isolate single cardiomyocytes with higher viability. The lineage and heterogeneity of various cells in the process of cardiac development can be comprehensively analyzed by scRNA-seq (Table 1). In 2016, a single-cell atlas of mouse embryonic and postnatal hearts was generated by two independent groups (DeLaughter et al., 2016; Li et al., 2016). They found that the homeobox gene Nkx-2.5 plays an important role in cardiac maturation. By a combination of scRNA-seq and Assay for Transposase-Accessible Chromatin using sequencing (ATAC-seq), Jia et al. (2018) studied mouse cardiac progenitor cells (CPCs) from E7.5 to E9.5 and found that the fate transition of cardiac progenitor cells is closely related to the chromatin state. This correlation depends on the changes of Isl1 and Nkx-2.5. Later on, Mesp1 was identified as another important factor for promoting transition from cardiac progenitors to cardiomyocytes (Lescroart et al., 2018). By single-cell transcriptomic analysis of Nkx-2.5 and Isl1 lineages, Xiong et al. (2019) presented a panoramic view of distinct CP differentiation hierarchies, supported the fact that the first and second heart fields (FHF and SHF, respectively) take different differentiation paths, and revealed the importance of Nkx-2.5 in Cxcr2-and Cxcr4-activated transcription in SHF.

**TABLE 1:** scRNA-seq and cardiovascular development.

Cardiovascular development	Technology	Species	Sample	Factor	Effect	Reference
Cardiac maturation	scRNA-seq	Mouse	Embryonic (E8.5, 9.5, and 10.5)	Nkx-2.5	Regulate the differentiation of cardiac myocytes	Li et al. (2016)
	scRNA-seq	Mouse	Embryonic and post-natal (E9.5 to P21)	Nkx-2.5	Maturation of distinct cardiac cell lineages	DeLaughter et al. (2016)
	scRNA-seq ATAC-seq	Mouse	Embryonic (E7.5∼E9.5)	Isl1, Nkx2.5	Regulation of heart development	Jia et al. (2018)
	scRNA-seq	Mouse	Embryonic (E6.75, E7.25)	Mesp1	Exit from the pluripotent state and the induction of the cardiovascular gene expression	Lescroart et al. (2018)
	scRNA-seq	Mouse	Embryonic (E7.75, E8.25, E8.75, and E9.25)	Nkx2.5, Cxcr2, and Cxcr4	Present a panoramic view of distinct CP differentiation hierarchies	Xiong et al. (2019)
	scRNA-seq	Human	hiPSC-derived cardiomyocytes (Days 0, 12, 24, and 90)	A series of factors	Differentiation or maturation stages in differentiating cardiomyocytes	Grancharova et al. (2021)
Spatial development	scRNA-seq	Mouse	Cardiac conduction system E16.5	Hcn4, Contactin 2	Validated conduction-specific markers	Goodyer et al. (2019)
	scRNA-seq	Mouse	Embryonic E7.75, E8.25, and E9.25	Hand2	Specific marker of outflow tract cells	de Soysa et al. (2019)
	scRNA-seq	Human	Embryonic/fetal hearts 4.5–10 weeks of fetal ages	LGR5	Novel cardiac progenitor marker	Sahara et al. (2019)
	scRNA-seq	Human	Cardiac cells from embryos ranging from 5 to 25 W of gestation	A series of factors	Investigated the differences in transcriptional profiles between humans and mice	Cui et al. (2019)
	scRNA-seq	Human	Embryonic 4.5–5, 6.5, and 9 post-conception weeks	A series of factors	Visualize 2D and 3D models of spatiotemporal gene expression patterns during heart development	Asp et al. (2019)

In addition, scRNA-seq contributes to the identification of factors responsible for the spatial development of the heart. Recently, uniquely expressed genes responsible for the development of the cardiac conduction system were identified in an embryonic mouse (Goodyer et al., 2019). Similarly, Hand2 was found to serve as a marker for outflow tract cells but not right ventricular cells, as proposed previously (de Soysa et al., 2019). On the contrary, the LGR5+ cardiac progenitor cells were found to be located in the outflow tract region of the human embryonic heart (Cui et al., 2019; Sahara et al., 2019). By a combination of spatial transcriptomics, Asp et al. (2019) generated a 3D single-cell transcriptional atlas of the embryonic human heart.

As for human vascular cells, Su et al. (2018) used scRNA-seq to study the dynamic lineage of coronary arteries and found that COUP-TF2 blocked arterial formation in the pre-arterial stage. Grancharova et al. (2021) used scRNA-seq to profile gene expression during the differentiation of human-induced pluripotent stem cells (hiPSCs) to cardiomyocytes, revealing genetic features which could identify stages of differentiation or maturation in differentiating cardiomyocytes. Currently, scRNA-seq has become an important tool for studying embryological development of the cardiovascular system. The applications of scRNA-seq will help dissect the mechanisms underlying common CVDs.

### scRNA-Seq and Cardiovascular Disease

scRNA-seq can comprehensively provide mRNA information of each cell in the heart and blood vessel in disease states, which can enable the discovery of new therapeutic targets for CVDs (Table 2). In a mouse model of ischemic heart disease, including myocardial infarction (MI), Zhang et al. (2019) observed limited but measurable myogenesis. However, Gladka et al. (2018) did not find any evidence of significant cardiac proliferation but observed excessive fibrosis after MI instead. They found that CKAP4 regulates fibroblast activation in damaged hearts and can be used as a marker for activated cardiac myofibroblasts. The heterogeneity of cardiac fibroblasts was further identified in a mouse MI heart in one subpopulation, Cthrc1 was shown to be involved in the scar healing process, and can, therefore, serve as a potential therapeutic target (Ruiz-Villalba et al., 2020). By scRNA-seq of infarcted and non-infarcted regions dissected from human heart samples, one novel transcription factor, AEBP1, was identified to regulate cardiac fibrosis during MI (Rao et al., 2021).

**TABLE 2:** scRNA-seq and cardiovascular disease.

Cardiovascular disease	Technology	Species	Sample	Factor	Effect	Reference
Myocardial infarction	scRNA-seq	Mouse	Infarcted myocardium	P53	Cardiomyocyte formation	Zhang et al. (2019)
Myocardial infarction	scRNA-seq	Mouse	Heart of mice collected 3 days after sham (control) or ischemia reperfusion surgery	CKAP4	Modulate fibroblast activation in the injured heart	Gladka et al. (2018)
Myocardial infarction	scRNA-seq and bulk RNA sequencing	Mouse, pig, and human	Hearts from healthy or infarcted models of mouse, pig, and human	CTHRC1	Cardiac healing	Ruiz-Villalba et al. (2020)
DCM and ICM	scRNA-seq	Human	Adult patients with DCM and ICM	AEBP1	Fibrosis regulator	Rao et al. (2021)
Myocardial hypertrophy	scRNA-seq	Mouse	Cardiomyocytes of mice exposed to pressure overload	ELK1, NRF1/2	Early heart failure	Nomura et al. (2018)
Cardiovascular fibrosis	scRNA-seq	Mouse	Hearts from a PlnR9C/+ mouse	IL-11	A potential therapeutic target of cardiovascular fibrosis	Schafer et al. (2017)
Chronic heart failure	scRNA-seq and scATAC-seq	Mouse	C57Bl/6J mice aged 8–10 weeks of transverse aortic constriction	MEOX1	Governs cellular plasticity in the fibroblast compartment during the pathogenesis	Alexanian et al. (2021)
Cardiac pacemaker	scRNA-seq	Mouse	Right atrium with the whole sinus node of male C57BL/6J mice	ion channels	Unique molecular make-up of the cardiac pacemaker	Linscheid et al. (2019)
Heart failure	scRNA-seq	Mouse	Pathological cardiac hypertrophy in a mouse model of pressure overload	ECs	Conservation across species	Ren et al. (2020)
Heart failure and recovery	scRNA-seq	Human	LV biopsies of two patients with HF	ACKR1+ ECs	Hub in cell–cell interactions	Wang et al. (2020)
Systematic vascular remodeling of hypertension	scRNA-seq	Human	Mesenteric artery and aortic artery from spontaneously hypertensive rats	a series of factors	aortic artery remodeling	Cheng et al. (2021)
atherosclerosis	scRNA-seq	Mouse and human	Mouse atherosclerotic aortas and human carotid artery atherosclerotic plaques	RA signaling	Cell phenotypic transformation	Pan et al. (2020)
Ascending thoracic aortic aneurysm	scRNA-seq	Human	Ascending aortic tissues from patients with ATAA	ERG	Maintain normal aortic function	Li et al. (2020)

Nomura et al. (2018) found that in pressure overload–induced cardiac hypertrophy and heart failure mouse models, ERK1/2 and NRF1/2 are involved in the regulation of early cardiac hypertrophy, while p53 is mainly involved in cardiac remodeling in the decompensated phase. Interestingly, Schafer et al. (2017) performed genome sequencing on the hearts of multiple fibrosis mice models and found that IL-11 is specifically expressed in activated cardiac fibroblasts and is a new specific marker for cardiac fibrosis. As a result, interfering with IL-11 gene expression or blocking the binding of IL-11 to receptors can effectively prevent and treat cardiac fibrosis, suggesting that IL-11 is an important therapeutic target. By combining scRNA-seq and scATAC-seq, Alexanian et al. (2021) recently identified that bromodomain and extraterminal (BET)–dependent regulation of MEOX1, a fibroblast-specific enhancer, controls fibroblast activation during chronic heart failure. In light of this, they suggested new therapeutic approaches targeting MEOX1 expression be developed instead of systemic BET inhibitors that have broad effects to alleviate the pathogenesis of fibrotic diseases.

Because of the large size of intact adult cardiomyocytes, researchers have used nuclei extracted from myocytes for scRNA-seq, so-called snRNA-seq. Linscheid et al. (2019) performed snRNA-seq of the sinoatrial node from the adult mouse heart and found that membrane clock–related channel proteins have a higher expression in the cardiomyocytes of the sinoatrial node than that of the atrium. More recently, Litviňuková et al. (2020) combined scRNA-seq and snRNA-seq methods to generate an atlas of adult human hearts. Another approach to carry out scRNA-seq of the adult human heart is to use FACS or a platform with a large-bore nozzle to pick a single myocyte. Using this technique, Ren et al. (2020) analyzed the spatiotemporal interplay among adult cardiomyocytes and non-cardiomyocytes in a pressure overload mouse model. They revealed that targeting macrophages by dapagliflozin, as well as TD139 and arglabin, could prevent cardiac hypertrophy from progressing to heart failure. For human heart failure, Wang et al. (2020) proposed that changes in heart function are most correlated with cardiac contractility and metabolism. They found that injection of ACKR1+-endothelial cells preserved cardiac function after injury.

Coming to blood vessels, Cheng et al. (2021) described the first cell atlas of resistant and conductive arteries in hypertensive rats. The dysregulated gene profile of individual cells during hypertensive vascular remodeling was characterized in artery type–specific and cell type–specific manners. For atherosclerosis, Gu et al. (2019) sequenced normal and apoE−/− mouse single cells and mapped the aortic adventitia, including endothelial cells, immune cells, adventitia mesenchymal cells, and other cell subgroups. It was revealed that the activation of macrophages triggers an inflammatory response in the blood vessels of apoE−/− mice, suggesting that the interaction between adventitial cells and immune cells is crucial in the early stages of atherosclerosis.

In addition, smooth muscle cells were reported to transition to macrophage- and fibrochondrocyte-like cells during atherosclerosis, a phenomenon regulated by the retinoic acid pathway (Pan et al., 2020). Recently, scRNA-seq was performed on patients with ascending thoracic aortic aneurysm (ATAA) and ascending aortic tissue of controls (Li et al., 2020). The authors not only described cellular composition and molecular alteration of the ascending aortic wall during the ATAA but also indicated the critical role of the ERG gene in the function of the aortic wall.

### scRNA-Seq and Cell-Based Therapy

In recent years, cardiovascular cells differentiated from human embryonic stem cells or hiPSCs have provided a new source of cells for repairing or regenerating the injured heart. Transplantation of cardiovascular cells was found to improve wall stress, cardiac metabolism, and contractile performance in a porcine heart model of MI (Xiong et al., 2013; Ye et al., 2014). Genetic modification of hiPSC-CMs to overexpress angiopoietin-1 or co-administration of hiPSC-CMs with thymosin β4 (Tb4) not only improved neovascularization of the infarcted myocardium but also improved hiPSC-CM viability, proliferation, and engraftment (Tan et al., 2021; Tao et al., 2021). Similarly, hiPSC-CMs overexpressing cyclin D2 promoted myocyte proliferation in both donor cells and recipient porcine hearts (Zhao et al., 2021).

However, the insufficient maturity of cardiomyocyte differentiation *in vitro* and the complexity of differentiated cardiomyocyte subsets are still unsolved questions (Lundy et al., 2013; Tan and Ye, 2018). By scRNA-seq of the mouse heart at different time points during differentiation and characterization of 1) CMs derived from stem cells and 2) CMs and ECs derived from a congenital heart disease mouse model, DeLaughter et al. (2016) distinguished and contrasted the developmental lineages of the cells of the left atria, primordial ventricle, and subsequently left and right ventricles at time points spanning embryonic to post-natal cardiac development and verified that the maturity of pluripotent stem cell–derived cardiomyocytes is positively correlated with the culture duration.

Ni et al. (2021) suggested the application of glucose starvation for stem cell differentiation to obtain homologous and mature cardiomyocytes. Meanwhile, Cho et al. (2017) investigated the difference between differentiated cardiomyocytes (either simply cultured or implanted into the heart of newborn rats) and adult cardiomyocytes by scRNA-seq and found that implantation into the heart of newborn rats could promote the maturation of stem cell–derived cardiomyocytes. This experiment not only established a stem cell–based adult arrhythmic right ventricular cardiomyopathy model but also indicated the reliability of cell-based therapy.

Furthermore, Monsanto et al. (2020) found scaffold-free three-dimensional (3D) microenvironments spontaneously formed by mesenchymal stem cells, endothelial progenitor cells, and c-Kit + cardiac interstitial cells cultured together, which was termed “CardioCluster,” in which the expression of stem cell–relevant factors, adhesion/extracellular matrix molecules, and cytokines was detected by scRNA-seq profiling. Afterward, Monsanto et al. injected the CardioCluster into murine MI models that were followed for 20 weeks and found that it improved cell retention and capillary density with preservation of cardiomyocyte size and long-term cardiac function. Despite cellular therapy for cardiac diseases being the focus of intensive research efforts, progress toward cardiac structural and functional recovery remains limited. Future studies should focus on developing different strategies to boost the potency of stem cell repair.

### scRNA-Seq and Bulk and Spatial RNA Sequencing

Although bulk RNA sequencing (bulk RNA-seq) is the basis for the development of scRNA-seq and spatial RNA sequencing (spRNA-seq), the latter two can offer transcriptional information at a single-cell level or near single-cell level (Yifan et al., 2020). In addition, data from scRNA-seq and spRNA-seq can be corroborated to reveal the spatial information of individual cells (Stark et al., 2019). For example, scRNA-seq can confirm and enrich the regional gene expression data obtained by spRNA-seq to generate a 3D atlas of the human embryonic heart (Asp et al., 2019). Similarly, by combining scRNA-seq and spRNA-seq, one subpopulation of cardiomyocytes overexpressing Nrf1 has been identified near the infarcted region of the heart (Cui et al., 2021). Thus, the activation of the Nrf1 pathway represents a new therapeutic approach for cardiac injury.

### Future Perspectives

scRNA-seq provides a powerful tool for studying cell heterogeneity, tracking cell dynamics, and subsequently finding new therapeutic strategies for CVDs. It has, thus, opened a fresh chapter in the field of modern cardiovascular research. With constant improvement in scRNA-seq technology and the combination of multiomics and spatiotemporal analysis, scRNA-seq will play a greater role in the field of cardiovascular research. In particular, the comprehensive study and analysis of genomics, epigenomics, and transcriptomics at the single-cell level holds promise for the elucidation of gene regulatory mechanisms at a single-cell resolution, contributes to individualized therapy of CVD in clinical practice, improves therapeutic efficacy and clinical outcomes, and facilitates the practice of precision medicine.
